# Changes in Outpatient Health Care Use After COVID-19 Infection Among Veterans

**DOI:** 10.1001/jamanetworkopen.2023.55387

**Published:** 2024-02-09

**Authors:** Paul L. Hebert, Kyle E. Kumbier, Valerie A. Smith, Denise M. Hynes, Diana J. Govier, Edwin Wong, Brystana G. Kaufman, Megan Shepherd-Banigan, Mazhgan Rowneki, Amy S. B. Bohnert, George N. Ioannou, Edward J. Boyko, Theodore J. Iwashyna, Ann M. O’Hare, C. Barrett Bowling, Elizabeth M. Viglianti, Matthew L. Maciejewski

**Affiliations:** 1 Center for Veteran-Centered and Value-Driven Care, Veterans Affairs Puget Sound Health Care System, Seattle, Washington; 2 Department of Health Systems and Population Health, University of Washington School of Medicine, Seattle; 3 VA Health Services Research & Development, Center for Clinical Management and Research, VA Ann Arbor Healthcare System, Ann Arbor, Michigan; 4 Center of Innovation to Accelerate Discovery and Practice Transformation (ADAPT), Durham Veterans Affairs Health Care System, Durham, North Carolina; 5 Department of Population Health Sciences, Duke University, Durham, North Carolina; 6 Department of Medicine, Duke University, Durham, North Carolina; 7 Center of Innovation to Improve Veteran Involvement in Care, Veterans Affairs Portland Health Care System, Portland, Oregon; 8 Health Management and Policy, Health Data and Informatics Program, Center for Quantitative Life Sciences, College of Health, Oregon State University, Corvallis; 9 School of Nursing, Oregon Health & Science University, Portland; 10 Duke-Margolis Center for Health Policy, Durham, North Carolina; 11 Department of Anesthesiology, University of Michigan, Ann Arbor; 12 Division of Gastroenterology, University of Washington, Seattle; 13 Epidemiologic Research and Information Center, Veterans Affairs Puget Sound Health Care System, Seattle, Washington; 14 Department of Epidemiology, University of Washington, Seattle; 15 Department of Medicine, University of Michigan Medical School, Ann Arbor; 16 School of Medicine, Johns Hopkins University, Baltimore, Maryland; 17 School of Public Health, Johns Hopkins University, Baltimore, Maryland; 18 Durham Veterans Affairs Geriatric Research Education and Clinical Center, Durham Veterans Affairs Medical Center, Durham, North Carolina

## Abstract

**Importance:**

The association of COVID-19 infection with outpatient care utilization is unclear. Many studies reported population surveillance studies rather than comparing outpatient health care use between COVID-19–infected and uninfected cohorts.

**Objective:**

To compare outpatient health care use across 6 categories of care (primary care, specialty care, surgery care, mental health, emergency care, and diagnostic and/or other care) between veterans with or without COVID-19 infection.

**Design, Setting, and Participants:**

In a retrospective cohort study of Veterans Affairs primary care patients, veterans with COVID-19 infection were matched to a cohort of uninfected veterans. Data were obtained from the Veterans Affairs Corporate Data Warehouse and the Centers for Medicare & Medicaid Services Fee-for-Service Carrier/Physician Supplier file from January 2019 through December 2022. Data analysis was performed from September 2022 to April 2023.

**Exposure:**

COVID-19 infection.

**Main Outcomes and Measures:**

The primary outcome was the count of outpatient visits after COVID-19 infection. Negative binomial regression models compared outpatient use over a 1-year preinfection period, and peri-infection (0-30 days), intermediate (31-183 days), and long-term (184-365 days) postinfection periods.

**Results:**

The infected (202 803 veterans; mean [SD] age, 60.5 [16.2] years; 178 624 men [88.1%]) and uninfected (202 803 veterans; mean [SD] age, 60.4 [16.5] years; 178 624 men [88.1%]) cohorts were well matched across all covariates. Outpatient use in all categories (except surgical care) was significantly elevated during the peri-infection period for veterans with COVID-19 infection compared with the uninfected cohort, with an increase in all visits of 5.12 visits per 30 days (95% CI, 5.09-5.16 visits per 30 days), predominantly owing to primary care visits (increase of 1.86 visits per 30 days; 95% CI, 1.85-1.87 visits per 30 days). Differences in outpatient use attenuated over time but remained statistically significantly higher at 184 to 365 days after infection (increase of 0.25 visit per 30 days; 95% CI, 0.23-0.27 visit per 30 days). One-half of the increased outpatient visits were delivered via telehealth. The utilization increase was greatest for veterans aged 85 years and older (6.1 visits, 95% CI, 5.9-6.3 visits) vs those aged 20 to 44 years (4.8 visits, 95% CI, 4.7-4.8 visits) and unvaccinated veterans (4.5 visits, 95% CI, 4.3-4.6 visits) vs vaccinated veterans (3.2 visits; 95% CI, 3.4-4.8 visits).

**Conclusions and Relevance:**

This study found that outpatient use increased significantly in the month after infection, then attenuated but remained greater than the uninfected cohorts’ use through 12 months, which suggests that there are sustained impacts of COVID-19 infection.

## Key Points

**Question:**

Do veterans infected with COVID-19 have different use of outpatient health care after infection than veterans who were not infected?

**Findings:**

In this cohort study including 202 803 veterans with COVID-19 and 202 803 matched uninfected veterans, differences in outpatient health care use between infected and uninfected patients were highly concentrated in the month immediately following infection, mostly because of primary care use, of which half was provided via telehealth. Use of outpatient health care decreased in the infected cohort but remained slightly greater than in the uninfected cohort 12 months after infection.

**Meaning:**

The adverse health outcomes of COVID-19 infection translated into increased use of outpatient services for up to 12 months after infection, suggesting persistent effects of post–COVID-19 conditions.

## Teaser

This cohort study examines outpatient health care use across 6 categories of care between US veterans with or without COVID-19 infection.

## Introduction

The COVID-19 pandemic strained health care systems worldwide and altered patient access to care. Systematic reviews^1,2,3^ of multinational evidence found a nearly universal decline in in-person outpatient health care use^4^ in the pandemic’s early stages, but a marked increase in the use of telehealth.^5,6,7,8^ Many of these prior health care utilization studies were population-level comparisons of health care use before and during the pandemic rather than comparisons of COVID-19–infected and uninfected cohorts. This confounds individual-level effects of COVID-19 on health care use with the pandemic’s systemic effects on health care delivery. Most studies that have assessed the individual-level effect of COVID-19 infection on health care use were based on pre-post designs following a positive COVID-19 test^9^ or compared patients with post–COVID-19 condition (PCC) with COVID-19–positive patients without long-term complications of COVID-19.^10,11^ To our knowledge, only 2 prior studies^12,13^ compared differences in health care use between infected and matched uninfected cohorts. Both studies found that COVID-19–infected patients had more health care encounters and outpatient visits than did patients without infection over 6 months^12^ or 12 months.^13^ Neither study examined outpatient health care use across specific phases of the clinical presentation of COVID-19, for specific components of outpatient care, or for specific modalities (in-person vs telehealth).

In this study, we compare outpatient health care use and its components (eg, primary care and specialty care) between matched cohorts of veterans with and without COVID-19 infection for 12 months before and 12 months after COVID-19 infection. We also characterize changes in outpatient use by modality and examine whether subgroups of veterans were disproportionately affected by COVID-19 infection. Comparing cohorts of patients who were exposed to the same economic, social, and health care–related burdens associated with the pandemic is necessary to isolate the effect of infection from the effect of the pandemic.^14^ By examining utilization beyond the acute period, this study also provides evidence about the potential impact on long-term health care use that may be due to longer term multisystemic symptoms and conditions. Finally, these analyses can inform which units within health systems may experience sustained demand in future pandemics and identify where telehealth may or may not offload demand for care.

## Methods

### Study Design and Population

**Figure 1.**
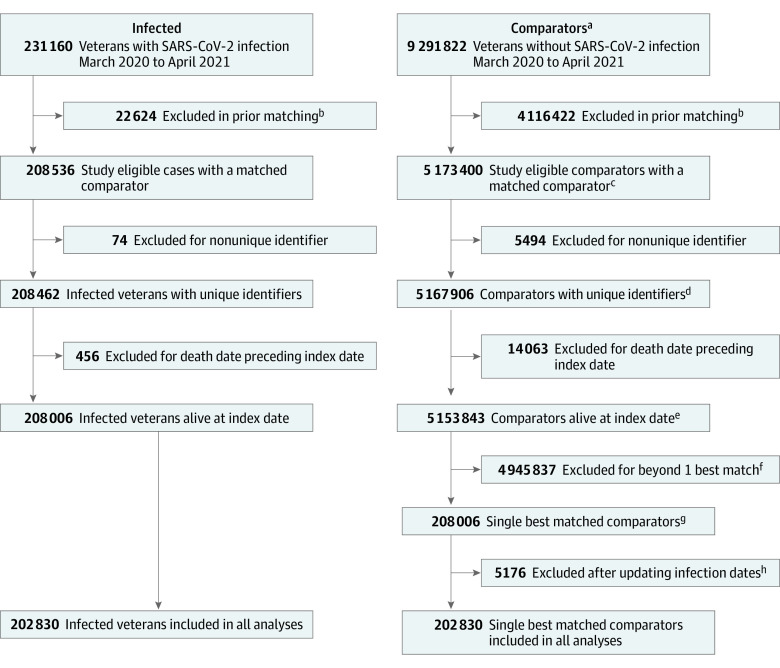
Derivation of COVID-19–Infected and Matched Uninfected Cohorts ^a^There were 1 222 272 comparators matched to more than 1 case. In the numbers presented in this figure, comparators who were matched to more than 1 infected case have been counted as many times as they appeared in the data set. The number of unique comparators at each step in the flow diagram are presented in the footnotes below. ^b^Those excluded in the prior matching included those with no Care Assessment Need score; no primary care practitioner; missing height, weight, or implausible value; age missing or implausible value; zip code missing or not in Washington, DC, or 50 states; had a Medicare COVID-19 diagnosis; and no suitable match. Those who became a case in the same month were also excluded among the comparators. ^c^The unique number of uninfected comparators matched to an infected case was 3 014 091. ^d^The unique number of matched comparators with unique identifiers was 3 012 243. ^e^The unique number of alive matched comparators at the index date was 3 006 856. ^f^The unique number of matched comparators who were dropped was 2 798 500. ^g^The unique number of matched comparators in initial best match was 201 070. Of the comparators in the initial best match, 6091 were matched to more than 1 infected case. ^h^There were 5052 cases (and their matched comparators) excluded from analysis because they had an updated infection date in a prior infection month. An additional 124 comparators (and their matched case) were excluded from analysis because they had an updated infection date in a prior month or the same month as their matched case.

In this retrospective cohort study, we compared outpatient health care use for veterans enrolled in the Veterans Affairs Health Care System (VA) with COVID-19 infection between March 1, 2020, and April 30, 2021, who were matched to uninfected comparators. The study sample included veterans who had an assigned VA primary care practitioner or who had at least 1 VA primary care clinic visit in the prior 2 years. Matching occurred in a 2-step process, as described elsewhere.^15^ First, each infected person was exactly matched to persons who remained uninfected as of the calendar month of their matched case’s infection date according to sex, state of residence, immunosuppressive medication use, and COVID-19 vaccination status. In the second step, veterans with COVID-19 infection were matched 1:1 to veterans without infection within their exact matched groups on the basis of a propensity score derived from a logistic regression containing 37 categorical and continuous electronic health record–based covariates representing demographic, comorbidity, and utilization characteristics that were expected to be confounders or risk factors for the outcomes of interest (Figure 1). We used 1:1 nearest neighbor matching with replacement. A veteran without COVID-19 could serve as a comparator for multiple veterans with COVID-19 infection.^16^ Covariate balance between cohorts was evaluated using standardized differences.^17^ The start of the postinfection period for an uninfected control was defined as the midpoint of the month of infection for the matched patient.

This cohort study was approved by the institutional review boards at the Seattle, Washington; Portland, Oregon; Durham, North Carolina; Ann Arbor, Michigan; and Palo Alto, California, VA medical centers, which waived the requirement to obtain informed consent because this was a retrospective study of electronic health records, in accordance with 45 CFR §46. This study follows the Strengthening the Reporting of Observational Studies in Epidemiology (STROBE) reporting guidelines for cohort studies.

### Health Care Use Outcomes

Outpatient use was obtained from the VA Corporate Data Warehouse and the Centers for Medicare & Medicaid Services Fee-for-Service Carrier/Physician Supplier file from January 2019 through December 2022, which captures outpatient health care use by Medicare-enrolled veterans. Prior research indicates that a substantial number of VA enrollees are dual users of care from both systems.^18,19,20,21^ Hospitalization dates from the Centers for Medicare & Medicaid Services Medicare Provider Analysis and Review file and Corporate Data Warehouse inpatient records were used to eliminate clinician visits that occurred during acute inpatient stays.

We applied a hierarchical classification algorithm to consistently define categories of VA and Medicare outpatient visits,^19,20,21^ according to procedure codes, practitioner specialty codes, and place of service codes present in both systems’ data. This enabled us to aggregate VA and Medicare use into an overall visit outcome and 6 mutually exclusive visit categories (primary care, specialty care, surgery care, mental health, emergency care, and diagnostic and/or other care, such as visits with nonmedical professionals like chaplains and calls to VA clinical call centers). A visit was defined as a unique combination of practitioner specialty code and date, so that a visit to a social worker, an internist, and a pharmacist on the same day would constitute 3 visits. VA visits with nurse practitioners were assigned to other categories of outpatient utilization, according to the VA clinic stop code, as in prior work.^22^ Medicare-covered visits with nurse practitioners were attributed to visit categories in the same proportion as VA visits, since Medicare claims lack VA clinic stop codes. Modality of care (in-person or telehealth) was assigned on the basis of procedure codes, practitioner modifier codes,^23,24^ and VA stop codes^22^ specific to telehealth.

### Statistical Analysis

Data analysis was performed from September 2022 to April 2023. We compared patient characteristics between infected and uninfected cohorts. The observation period for health care use was divided into a 52-week preinfection period and 3 postinfection periods that correspond to the clinical presentation of COVID-19^25^: (1) a 0- to 30-day peri-infection period, (2) a 31- to 182-day intermediate postinfection period, and (3) a 183- to 365-day long-term postinfection period. A plot of weekly visits over the postinfection period provided visual evidence that these cutoffs were reasonable for modeling health care use. Negative binomial models using generalized estimating equations with an unstructured covariance matrix and robust SEs were used to estimate the effect of infection in each time period, adjusting for infection status, 3 time-period fixed effects, and interactions of infection and time-period fixed effects.

Censoring occurred when 1 of the matched pairs of infected and uninfected veterans died or when the uninfected veteran became infected. To maintain balance in veteran characteristics, we included visits in each follow-up period only for days that both members of a matched pair were uncensored and included an offset to account for number of days uncensored. Three separate models were estimated for each modality (in-person, telehealth, and combined telehealth plus in-person) for each category of outpatient visits and the sum of all outpatient visits. Difference-in-differences estimates were generated to account for time-invariant unobserved confounding. To facilitate interpretation and to account for the difference in exposure times among postinfection time periods, we normalized estimated differences in numbers of visits between infected and uninfected cohorts into a visit rate per 30 days.

Veteran characteristics were chosen for subgroup analyses according to differences reported in the clinical severity of COVID-19 infection,^26,27,28^ including age groups (20-44, 45-54, 55-64, 65-74, 75-84, and ≥85 years), sex, self-reported race, self-reported Hispanic ethnicity, smoking status, immunosuppression at index date, vaccination status at index date, rural or urban residence, comorbidity burden (defined as Gagne score^29^quartiles), and the pandemic wave in which the infection occurred.^30^ Race and ethnicity in the VA Corporate Data Warehouse are collected through self-identification either at enrollment or at a health care encounter and are included in this study for descriptive purposes and because outpatient visits are known to vary by race and ethnicity. We estimated separate models for total visits for each subgroup with main effects and all 2-way and 3-way interactions of infection, subgroup membership, and time-period fixed effects. For all subgroups except age, these models also included main effects for Medicare eligibility because standardized mean differences for Medicare eligibility were greater than 0.1 between infected and uninfected comparators. Models for comorbidity subgroups additionally controlled for smoking status, and models for vaccination subgroups additionally controlled for age and risk of hospitalization or death as measured by the Care Assessment Need^31^ score because of standardized mean differences greater than 0.1 in these variables for these subgroups.

In sensitivity analyses, we dropped visits on the date of infection for infected and uninfected patients to address the possibility that visits were scheduled solely for COVID-19 testing. We also calculated the e-value^32^ to assess the extent to which the results were subject to confounding. Statistical significance was set a priori at 2-sided *P* < .05 for analyses and conducted in SAS statistical software version 9.4 (SAS Institute) and Stata statistical software version 17 (StataCorp).^33^

## Results

### Patient Characteristics in the Matched Cohorts

The cohort of 202 830 veterans with COVID-19 infection (mean [SD] age, 60.5 [16.2] years; 178 624 men [88.1%]; 1907 American Indian/Alaska Native [0.9%]; 2015 Asian [1.0%]; 46 524 Black or African American [22.9%]; 19 796 Hispanic [9.8%]; 1904 Native Hawaiian or other Pacific Islander [0.9%]; 136 035 White, [67.1%]) were well matched to 202 830 veterans without infection (mean [SD] age, 60.4 [16.5] years; 178 624 men [88.1%]; 1852 American Indian/Alaska Native [0.9%]; 2070 Asian [1.0%]; 46 838 Black or African American [23.1%]; 19 196 Hispanic [9.5%]; 1902 Native Hawaiian or other Pacific Islander [0.9%]; 135 916 White [67.0%]) across all sociodemographic and measured health characteristics (Table). Most veterans lived in urban areas (145 341 [71.7%] with COVID-19 infection and 145 024 [71.5%] without infection), approximately 13% were current smokers (25 455 [12.5%] with infection and 25 812 [12.7%] without infection), and a majority were Medicare enrolled (105 515 [52.0%] with infection and 113 358 [55.9%] without infection). The most prevalent conditions were hypertension (124 175 veterans with COVID-19 [61.2%] and 123 933 uninfected veterans [61.1%]), diabetes (69 945 veterans with COVID-19 [34.5%] and 68 891 uninfected veterans [34.0%]), and major depression (65 502 veterans with COVID-19 [32.3%] and 65 491 uninfected veterans [32.3%]). A minority were immunosuppressed (19 789 veterans with COVID-19 [9.8%] and 19 789 uninfected veterans [9.8%]) or in a community living center (2081 veterans with COVID-19 [1.0%] and 1830 uninfected veterans [0.9%]), which is the VA’s term for a nursing home.

**Table.** Baseline Characteristics of Matched Cohorts of Veterans With and Without COVID-19 Infection

Variable	Participants, No. (%)^a^		SMD
	With COVID-19 infection (n = 202 830)	Without COVID-19 infection (n = 202 830)^b^	
Age, mean (SD), y	60.5 (16.2)	60.4 (16.5)	0.004
Body mass index, mean (SD)^c^	31.4 (6.3)	31.3 (6.6)	0.015
Sex			
Female	21 392 (10.5)	21 392 (10.5)	0.000
Male	178 624 (88.1)	178 624 (88.1)	
Unknown	2814 (1.4)	2814 (1.4)	
Race			
American Indian/Alaska Native	1907 (0.9)	1852 (0.9)	0.006
Asian	2015 (1.0)	2070 (1.0)	
Black or African American	46 524 (22.9)	46 838 (23.1)	
Native Hawaiian or other Pacific Islander	1904 (0.9)	1902 (0.9)	
White	136 035 (67.1)	135 916 (67.0)	
Multiple races	1921 (0.9)	1892 (0.9)	
Missing	12 524 (6.2)	12 360 (6.1)	
Hispanic ethnicity			
Yes	19 796 (9.8)	19 196 (9.5)	0.010
No	176 121 (86.8)	176 673 (87.1)	
Missing	6913 (3.4)	6961 (3.4)	
Rurality			
Urban	145 341 (71.7)	145 024 (71.5)	0.003
Not urban (including missing)	57 489 (28.3)	57 806 (28.5)	
Smoking status			
Current	25 455 (12.5)	25 812 (12.7)	0.011
Former	85 789 (42.3)	85 757 (42.3)	
Never	79 797 (39.3)	79 962 (39.4)	
Missing	11 789 (5.8)	11 299 (5.6)	
CDC high-risk conditions			
Coronary heart disease	58 044 (28.6)	57 329 (28.3)	0.008
Cancer	37 047 (18.3)	36 797 (18.1)	0.003
Chronic kidney disease	46 077 (22.7)	45 357 (22.4)	0.008
Congestive heart failure	21 388 (10.5)	20 744 (10.2)	0.010
Pulmonary	44 695 (22.0)	44 623 (22.0)	0.001
Dementia	10 313 (5.1)	9883 (4.9)	0.010
Diabetes	69 945 (34.5)	68 891 (34.0)	0.011
Hypertension	124 175 (61.2)	123 933 (61.1)	0.002
Liver disease	14 894 (7.3)	14 668 (7.2)	0.004
Sickle cell	378 (0.2)	351 (0.2)	0.003
Transplant	670 (0.3)	627 (0.3)	0.004
Stroke or cerebrovascular disease	12 544 (6.2)	12 328 (6.1)	0.004
Substance use disorder	25 145 (12.4)	25 376 (12.5)	0.003
Anxiety	45 859 (22.6)	45 701 (22.5)	0.002
Bipolar disorder	7804 (3.8)	7772 (3.8)	0.001
Major depression	65 502 (32.3)	65 491 (32.3)	0.000
PTSD	51 550 (25.4)	51 798 (25.5)	0.003
Schizophrenia	4563 (2.2)	4476 (2.2)	0.003
CDC high-risk conditions, mean (SD), No.	2.29 (1.94)	2.27 (1.92)	0.011
Mental health conditions (anxiety, bipolar disorder, major depression, PTSD, schizophrenia), mean (SD), No.	0.86 (1.06)	0.86 (1.05)	0.000
Gagne score, mean (SD)	1.43 (2.34)	1.40 (2.24)	0.013
Inpatient admissions in prior year, mean (SD), No.	0.4 (1.2)	0.4 (1.3)	0.000
Primary care visits in prior year, mean (SD), No.	8.5 (9.7)	8.3 (10.6)	0.026
Specialty care visits in prior year, mean (SD), No.	13.9 (14.1)	13.5 (15.6)	0.027
Mental health visits in prior year, mean (SD), No.	7.8 (22.6)	7.8 (21.9)	0.001
Immunosuppressed in prior 24 mo	19 789 (9.8)	19 789 (9.8)	0.000
Residing in community living center at index date	2081 (1.0)	1830 (0.9)	0.013
Nosos risk adjustment score			
Missing	4591 (2.3)	5341 (2.6)	0.037
Category 1 (>0 to ≤0.417)	5550 (2.7)	5109 (2.5)	
Category 2 (>0.417 to ≤0.471)	9335 (4.6)	8921 (4.4)	
Category 3 (>0.471 to ≤0.534)	12 137 (6.0)	11 943 (5.9)	
Category 4 (>0.534 to ≤0.611)	14 827 (7.3)	14 704 (7.2)	
Category 5 (>0.611 to ≤0.707)	17 280 (8.5)	17 632 (8.7)	
Category 6 (>0.707 to ≤0.829)	20 045 (9.9)	20 437 (10.1)	
Category 7 (>0.829 to ≤0.998)	22 851 (11.3)	23 635 (11.7)	
Category 8 (>0.998 to ≤1.259)	26 237 (12.9)	26 515 (13.1)	
Category 9 (>1.259 to ≤1.805)	30 544 (15.1)	30 679 (15.1)	
Category 10 (>1.805 to infinity)	39 433 (19.4)	37 914 (18.7)	
Care Assessment Need score			
Missing	3900 (1.9)	4629 (2.3)	0.034
Category 1 (0-20)	33 770 (16.6)	33 307 (16.4)	
Category 2 (21-40)	31 209 (15.4)	31 235 (15.4)	
Category 3 (41-60)	37 235 (18.4)	37 829 (18.7)	
Category 4 (61-80)	45 479 (22.4)	45 973 (22.7)	
Category 5 (81-90)	29 801 (14.7)	29 758 (14.7)	
Category 6 (91-99)	21 436 (10.6)	20 099 (9.9)	
Vaccinated >2 wk before index			
No (January 2021 or later)	65 714 (32.4)	65 714 (32.4)	0.000
Yes (January 2021 or later)	3035 (1.5)	3035 (1.5)	
Not applicable (before January 2021)	134 081 (66.1)	134 081 (66.1)	
Index infection month			
March 2020	2333 (1.2)	2333 (1.2)	0.000
April 2020	6631 (3.3)	6631 (3.3)	
May 2020	4211 (2.1)	4211 (2.1)	
June 2020	6872 (3.4)	6872 (3.4)	
July 2020	14 667 (7.2)	14 667 (7.2)	
August 2020	8145 (4.0)	8145 (4.0)	
September 2020	6629 (3.3)	6629 (3.3)	
October 2020	11 991 (5.9)	11 991 (5.9)	
November 2020	30 338 (15.0)	30 338 (15.0)	
December 2020	42 264 (20.8)	42 264 (20.8)	
January 2021	35 268 (17.4)	35 268 (17.4)	
February 2021	15 054 (7.4)	15 054 (7.4)	
March 2021	9649 (4.8)	9649 (4.8)	
April 2021	8778 (4.3)	8778 (4.3)	
Distance to nearest Veterans Affairs medical center, mean (SD), miles	35.6 (36.5)	35.7 (35.1)	0.004
Reason for Medicare entitlement			
Old age and survivor’s insurance	59 989 (29.6)	62 363 (30.7)	0.093
Disability insurance benefits	9519 (4.7)	12 587 (6.2)	
End-stage kidney disease	36 007 (17.8)	38 408 (18.9)	
Not in Medicare	97 315 (48.0)	89 472 (44.1)	
Enrolled in Medicare Advantage			
Yes	55 343 (27.3)	59 115 (29.1)	0.078
No	50 172 (24.7)	54 243 (26.7)	
Not in Medicare	97 315 (48.0)	89 472 (44.1)	

Abbreviations: CDC, Centers for Disease Control and Prevention; PTSD, posttraumatic stress disorder; SMD, standardized mean difference.

^a^ A total of 196 235 unique Veteran comparators were used for analysis. Of the 202 830 comparators used for analysis, 5793 were matched to more than 1 infected case.

^b^ The unique number of matched comparators used for analysis was 196 235. Of the comparators used for analysis, 5793 were matched to more than 1 infected case.

^c^ Body mass index is calculated as weight in kilograms divided by height in meters squared.

### Analyses of Outpatient Use

**Figure 2.**
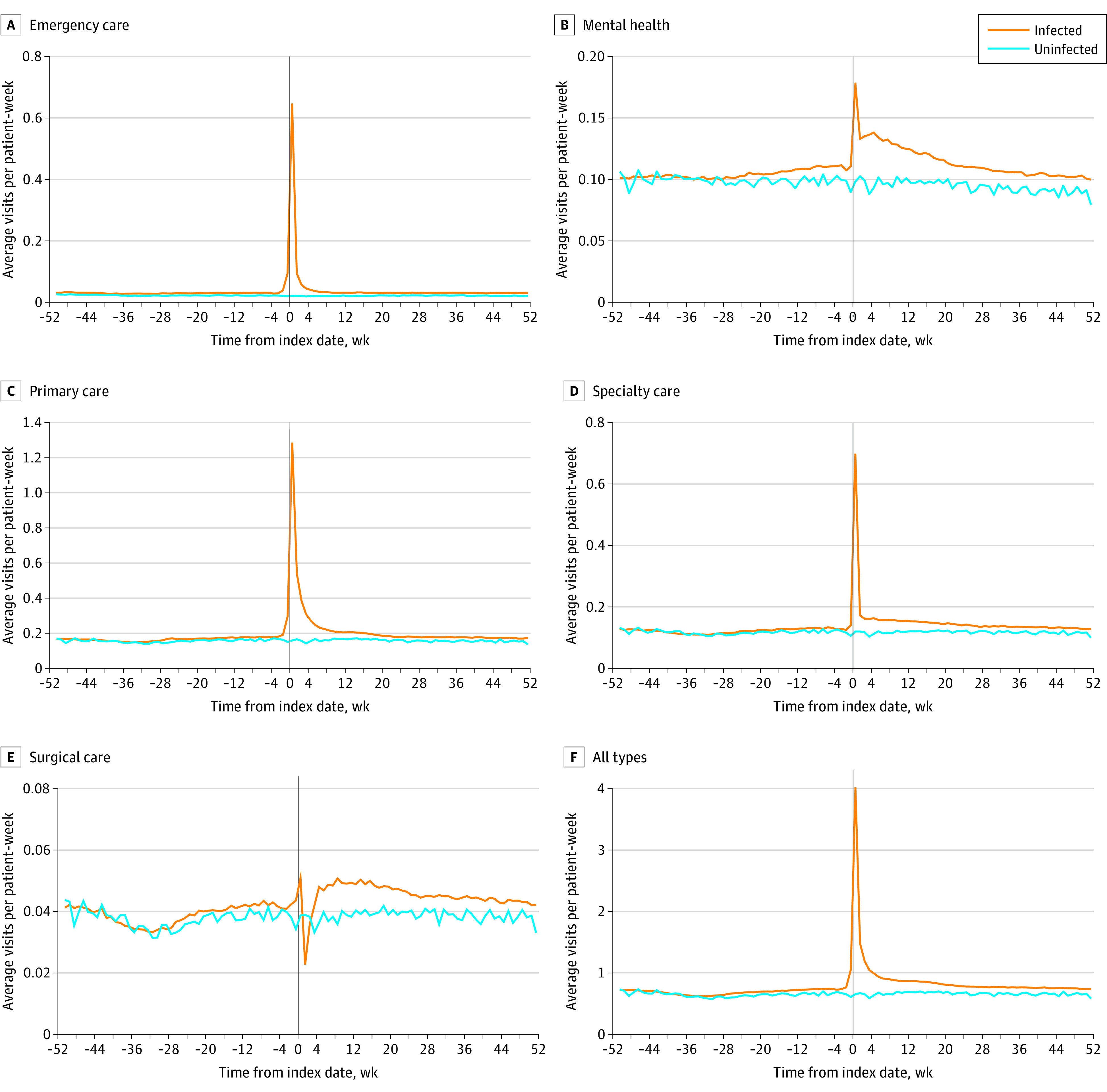
Unadjusted Weekly Outpatient Visit Use for COVID-19–Infected and Uninfected Veterans, by Category of Outpatient Visit, for the 52 Weeks Before and 52 Weeks After Infection

Unadjusted counts of outpatient visits (Figure 2) showed only small differences for those with and without COVID-19 infection (0.70 visits per week for the infected cohort vs 0.65 visits per week for the uninfected cohort in the preinfection period, 52 weeks before infection). There was an increase in visits for veterans with COVID-19 infection in the week of infection (4.02 visits vs 0.65 visits the previous week) immediately following the infection, with an attenuation after the first 4 weeks following infection (from 0.83 to 0.66 visits). Primary care use among infected patients deviated from uninfected patients in the week before infection, which might reflect the veterans’ discussing COVID-19–related symptoms with clinicians by telephone before receiving a positive test.

**Figure 3.**
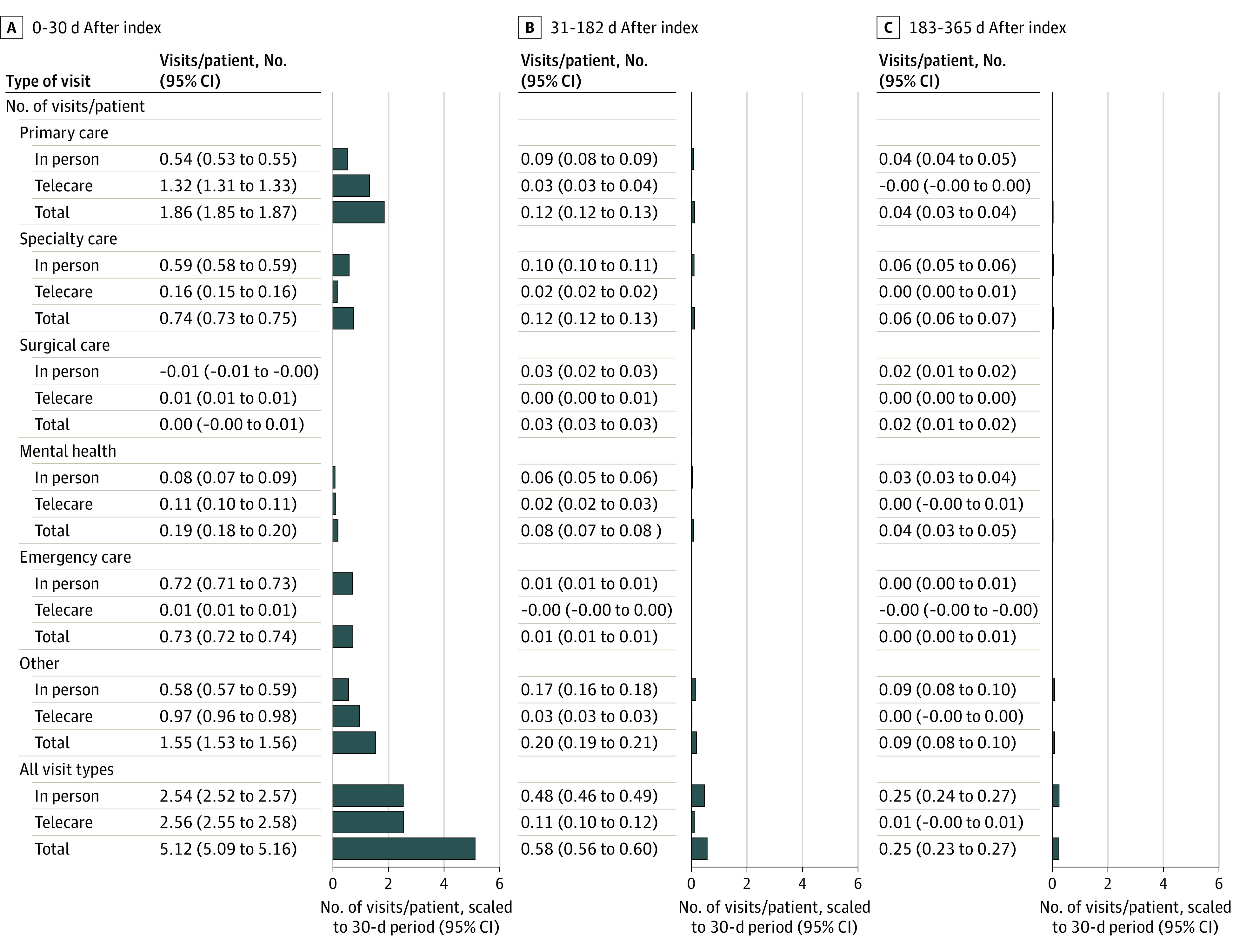
Difference-in-Difference Estimates of the Association of COVID-19 Infection With Outpatient Visits Over 30 Days Across 3 Postinfection Periods, by Category and Mode (Telecare vs In-Person) of Outpatient Visit

The adjusted difference-in-difference analyses (Figure 3) were consistent with the weekly unadjusted results. For outpatient visits overall, infected patients had a significant increase in visits to 5.12 visits per person (95% CI, 5.09-5.16 visits) in the 30 days after infection compared with matched uninfected patients, of which 50% were in person and 50% were via telehealth. The differential number of outpatient visits then declined to 0.58 visits (95% CI, 0.56-0.60 visits) per 30 days in the intermediate period (31-182 days after infection) and 0.25 visits (95% CI, 0.23-0.27 visits) in the long-term period (183-365 days after infection). Over 1 year, an infected veteran had 9.59 more visits (95% CI, 9.38-9.80 visits) than a veteran who remained uninfected for that year.

The 6 components of overall outpatient visits showed a similar short-term increase and subsequent attenuation, with the exception of surgical visits, which remained similar to preinfection trends. Although the short-term increase in outpatient visits was observed in the other 5 components, they differed in whether the visit increase was related to in-person care or telehealth. Visit increases for infected patients occurred for in-person specialty care (0.74 more specialty visits; 95% CI, 0.73-0.75 visits; 0.59 in-person visits) and emergency care (0.73 visits for emergency care; 95% CI, 0.72-0.74 visits; 0.72 in-person visits) in the month after infection but not for telehealth. On the other hand, visit increases occurred more via telehealth for primary care, mental health care, and other care in the month after infection, which persisted. There were 1.86 more primary care visits (95% CI, 1.85-1.87 visits) overall in the month after infection for infected patients, with 1.32 of these visits (95% CI, 1.31-1.33 visits) via telehealth. There were 0.19 (95% CI, 0.18-0.10 visits) more mental health visits in the month after infection, with 0.11 of these visits (95% CI, 0.10-0.11 visits) occurring via telehealth.

Excluding visits that occurred on the day of a positive test (eFigure and eTable in Supplement 1) yielded similar patterns of visits, indicating that care related to the COVID-19 test itself was not responsible for elevated visits in the 30 days after infection. When evaluating the potential for unobserved confounding via e-value, the e-value for the perioperative period was 4.88, meaning that the effect size of a confounder had to be very large (4.88 or greater) to explain away the estimated effect. The corresponding e-values for the intermediate and long-term periods were both 1.63.

### Subgroup Analyses

**Figure 4.**
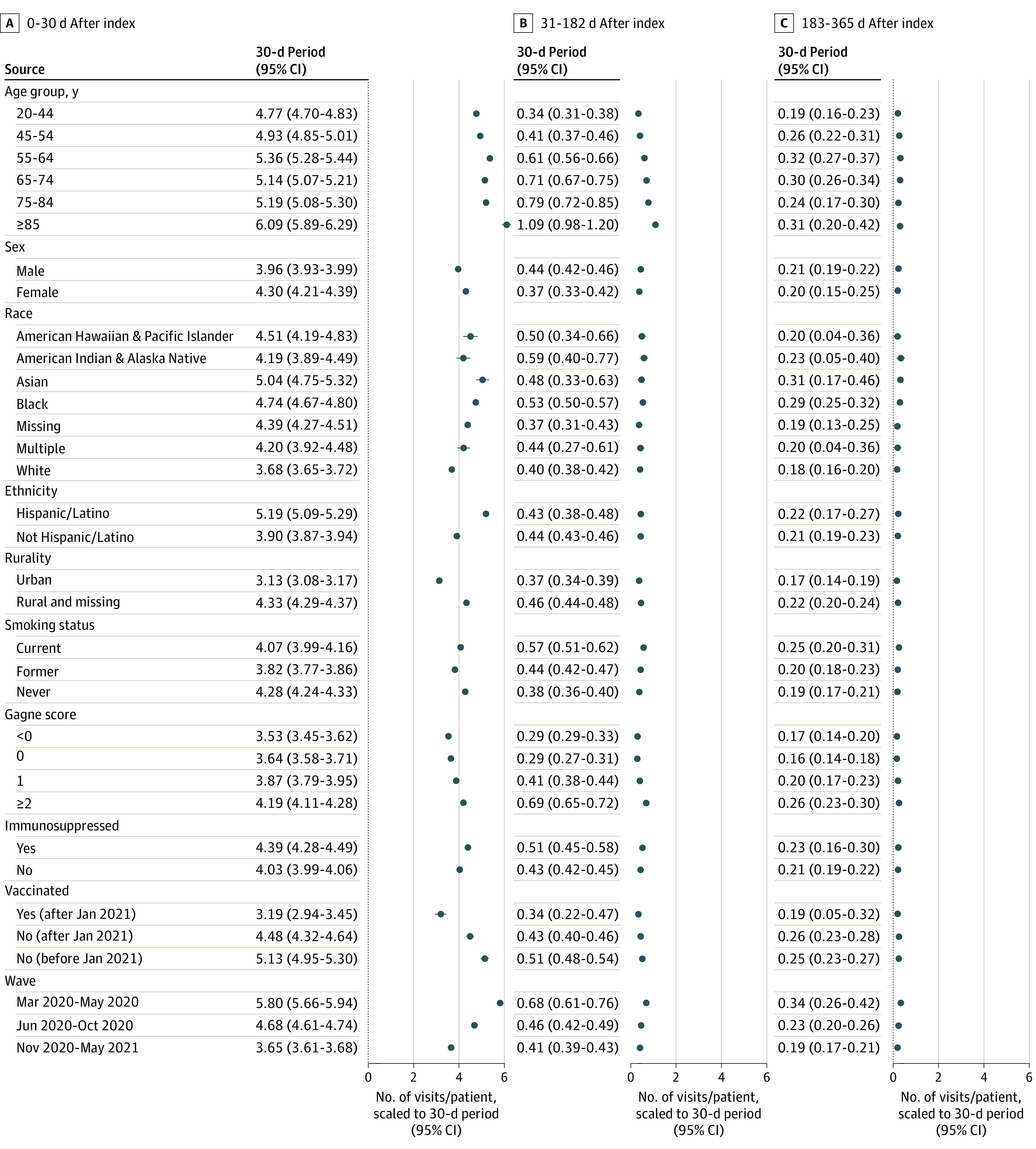
Difference-in-Difference Estimates of the Association of COVID-19 Infection With Total Outpatient Visits Over 30 Days Across 3 Postinfection Periods, by Patient Subgroups The estimates are the difference between infected patients and uninfected patients in the difference from baseline to each of the defined follow-up periods, for each of the defined subgroups. Models included fixed effects for Medicare eligibility, which was imbalanced between infected and uninfected cohorts within many subgroups. Models for comorbidity subgroups additionally controlled for smoking status, and models for vaccination subgroups additionally controlled for age and risk of hospitalization or death as measured by the Care Assessment Need score because of standardized mean differences >0.1 in these variables for these subgroups.

Results from prespecified subgroup analyses (Figure 4) followed similar health care utilization patterns as the main analysis. The visit increases for infected patients were markedly larger for veterans aged 85 years and older (6.1 visits; 95% CI, 5.9-6.3 visits) vs those aged 20 to 44 years (4.8 visits, 95% CI, 4.7-4.8 visits), veterans with greater comorbidity burden in the fourth quartile (4.2 visits; 95% CI, 4.1-4.3 visits) vs those with comorbidity burden in the first quartile (3.5 visits; 95% CI, 3.4-3.6 visits), unvaccinated veterans (4.5 visits; 95% CI, 4.3-4.6 visits) vs vaccinated veterans (3.2 visits; 95% CI, 3.4-4.8 visits), and rural veterans (4.3 visits; 95% CI, 4.3-4.4 visits) vs urban veterans (3.1 visits; 95% CI, 3.1-3.2 visits). Hispanic veterans had a larger peri-infection visit increase (5.2 visits; 95% CI, 5.1-5.3 visits) than non-Hispanic veterans (3.9 visits; 95% CI, 3.9-3.9 visits). The largest effects of COVID-19 infection on visits were in the first wave of the pandemic from March to May 2020 (5.8 visits; 95% CI, 5.7-5.9 visits), but the effects persisted in wave 2 from June to October 2020 (4.7 visits; 95% CI, 4.6-4.7 visits), and wave 3 from November 2020 to May 2021 (3.6 visits; 95% CI, 3.6-3.7 visits).

## Discussion

This cohort study of outpatient health care use between matched cohorts of veterans with and without COVID-19 infection in the year before after COVID-19 infection documented in VA and Medicare records yielded 3 important findings. First, we found marked increases in visits for veterans with COVID-19 infection compared with uninfected comparators in the 30 days following infection across all categories of outpatient visits, except for surgical visits. Primary care was the largest contributor to this overall outpatient visit increase, representing 36% (1.86 visits/5.12 visits overall) of the increase in the first month after infection, half of which was delivered via telehealth. The increased telehealth use for primary care and mental health care has been sustained since 2022, suggesting that the pandemic induced a modality switch for certain care types that may be more patient centered and clinically appropriate for patients with an infectious disease. Future research should examine the extent to which the sustained increase in telehealth visits represents patient preferences, practitioner or health system transition in modality, or other factors. These results reinforce the value in determining which types of care may be equally effective when delivered by telehealth and which types of care are best delivered in person.

Second, outpatient use by veterans with COVID-19 infection decreased after the first 30 days, but remained significantly higher than longer-term visits in uninfected veterans. Although the difference in COVID-19–attributable visits was less than 1 visit per 30 days, this represents an additional 904 000 visits for the 202 000 infected veterans from day 31 to 365 postinfection when multiplying the number of infected patients in our sample by the marginal differences in outpatient visits. Sustained higher outpatient use may be due to patients with PCC, which merits exploration in future work and has potential implications for VA workforce planning. Third, subgroup analyses found that the short-term visit increase following COVID-19 infection was greater for older veterans,^28^ veterans with a high comorbidity burden, Hispanic veterans,^27^ rural veterans, and veterans who were not vaccinated.^26^ Improved access to telehealth for these more vulnerable veterans may benefit them and their caregivers, if telehealth obviates the need for caregivers to provide veterans with travel for care.

The largest effects of COVID-19 infection on visits were in the first wave of the pandemic from March to May 2020 when COVID-19 testing was scarce, consistent with the hypothesis that the most acutely ill veterans were tested for COVID-19 and brought to the clinic in the earliest wave. The association of COVID-19 and outpatient visits persisted in wave 2 from June to October 2020 and wave 3 from November 2020 May 2021, which suggests testing and access to care alone were not responsible for the elevated visits.

These findings contrast somewhat to a prior analysis^12^ that also found elevated visits for COVID-19–positive patients 6 months after infection, but these elevated rates were due entirely to higher telehealth visits immediately after infection. Outpatient visits were lower overall for COVID-19–positive patients than uninfected patients between 2 weeks and 6 months after infection, and lower over the entire 6-month period for COVID-19–positive patients for in-person outpatient visits. The lower visit rates in that study might be due to sample restriction to patients who were alive for the entire 6-month follow-up period, which would exclude the most ill patients with COVID-19 and may diminish the visit difference compared with the uninfected cohort, or might be due to differences in VA vs non-VA care delivery. Our findings are more consistent with results from a retrospective cohort study of Canadian patients with COVID-19 infection matched 1:1 to uninfected patients^13^ that found elevated outpatient visit rates for infected patients over a 12-month follow-up period. Future work is needed to explore inpatient use following COVID-19 infection and outpatient use of patients identified with PCC.

### Limitations

We acknowledge several limitations. These analyses may be subject to unobserved confounding because patients were not randomized and we could not exactly match on every available characteristic associated with infection and health care utilization. A difference-in-differences design was used as an additional confounding control. Some patients who had COVID-19 but were not tested in the VA might have been misclassified. As a result, the estimates reported here are potentially biased toward the null. In addition, we lacked data on visits not provided by VA or paid for by Medicare. The effect of infection on visits in the peri-infection period maybe an underestimate if symptomatic veterans have virtual visits with practitioners before testing positive. In addition, telehealth visit counts are assumed to be correctly coded by modality but were not validated. Furthermore, these results may not generalize beyond veterans identified with COVID-19 in VA and obtaining care in VA or Medicare.

## Conclusions

In this retrospective cohort study, outpatient use for veterans enrolled in VA for all categories of care except surgical care increased in the 30 days after infection, then decreased but remained greater than uninfected cohorts’ use for at least 12 months after infection. These results suggest long-term impacts of COVID-19 infection on outpatient health care use and highlight the important role of telehealth in the delivery of primary care to veterans with COVID-19 infections.

## Notes

Supplement 1. **eFigure.** Difference-in-Difference Estimates of the Effect of COVID-19 Infection on Outpatient Visits Over 30 Days Across Three Post-Infection Time Periods, by Category and Mode (Telecare vs In-Person) of Outpatient Visit, Excluding Visits on the Day of Infection **eTable.** Final List of Covariates for Matching

Supplement 2.
VA COVID-19 Observational Research Collaboratory (CORC) Group Members

Supplement 3.
Data Sharing Statement
