# Supplementary material for: Changes in Outpatient Health Care Use After COVID-19 Infection Among Veterans
Source: JAMA Netw Open. 2024 Feb 9;7(2):e2355387. doi: 10.1001/jamanetworkopen.2023.55387 (PMC10858406; doi:10.1001/jamanetworkopen.2023.55387)
Supplement: Supplement 1. — eFigure. Difference-in-Difference Estimates of the Effect of COVID-19 Infection on Outpatient Visits Over 30 Days Across Three Post-Infection Time Periods, by Category and Mode (Telecare vs In-Person) of Outpatient Visit, Excluding Visits on the Day of Infection eTable. Final List of Covariates for Matching [file jamanetwopen-e2355387-s001.pdf]

Supplemental Online Content

Hebert PL, Kumbier KE, Smith VA, et al; VA COVID-19 Observational Research Collaboratory (CORC). Changes in outpatient health care use after COVID-19 infection among veterans. *JAMA Netw Open*. 2024;7(2):e2355387. doi:10.1001/jamanetworkopen.2023.55387

eFigure. Difference-in-Difference Estimates of the Effect of COVID-19 Infection on Outpatient Visits Over 30 Days Across Three Post-Infection Time Periods, by Category and Mode (Telecare vs In-Person) of Outpatient Visit, Excluding Visits on the Day of Infection
eTable. Final List of Covariates for Matching

This supplemental material has been provided by the authors to give readers additional information about their work.

© 2024 Hebert PL et al. *JAMA Network Open.*

eFigure: Difference-in-Difference Estimates of the Effect of COVID-19 Infection on Outpatient Visits Over 30 Days Across Three Post-Infection Time Periods, by Category and Mode (Telecare vs In-Person) of Outpatient Visit, Excluding Visits on the Day of Infection

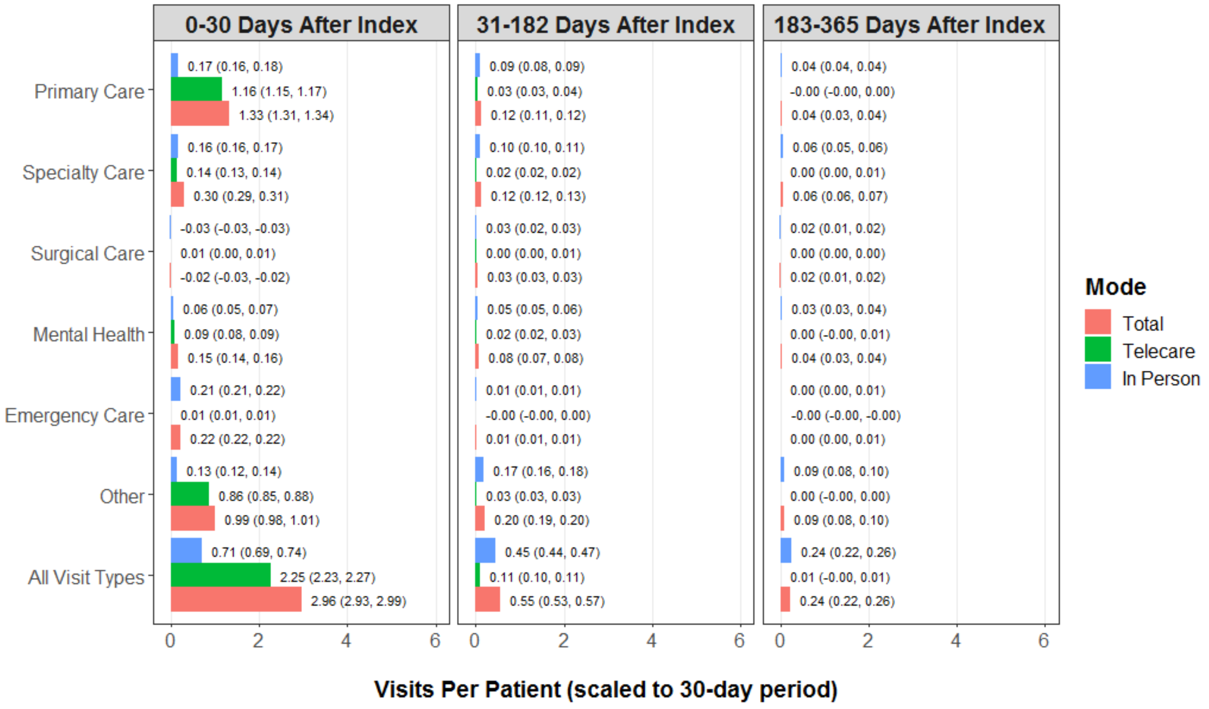

© 2024 Hebert PL et al. *JAMA Network Open.*

eTable. Final List of Covariates for Matching

Final Matching Specification Used
Sex
Use of immunosuppressive medication
Age
Race
Ethnicity
CAN comorbidity score (7 categories)
Nosos comorbidity score (11 categories)
Gagne comorbidity score
Indicator of CDC high-risk conditions (Cancer other than non-metastatic skin cancer)
Indicator of CDC high-risk conditions (COPD/asthma/interstitial lung disease/cystic fibrosis/pulmonary)
Indicator of CDC high-risk conditions (hypertension)
Indicator of CDC high-risk conditions (diabetes)
Indicator of CDC high-risk conditions (Dementia)
Indicator of CDC high-risk conditions (CAD)
Indicator of CDC high-risk conditions (Liver disease)
Indicator of CDC high-risk conditions (Pregnancy)[1]
Indicator of CDC high-risk conditions (Sickle cell/ thalassemia)
Smoking status (4 categories)
Indicator of CDC high-risk conditions (Solid organ or blood stem cell transplant)
Indicator of CDC high-risk conditions (Stroke or cerebrovascular disease)
Indicator of CDC high-risk conditions (CKD)
Indicator of CDC high-risk conditions (CHF)
Indicator of CDC high-risk conditions (SUD)
Count of CDC high-risk conditions
Anxiety Diagnosis
PTSD Diagnosis
Bipolar Diagnosis
Schizophrenia Diagnosis
Major depression Diagnosis
Count of mental health conditions
Body Mass Index (BMI)
Community Living Center resident
admissions (VA + non-VA)
VA primary care visits
VA specialty care visits
VA mental health care visits
State of residence
Distance to nearest VA medical center
Rurality of residence (urban, rural)
Vaccinated for SARS-CoV-2 (January-April 2021)

Abbreviations: CAN: Care Assessment Need; CDC: Centers for Disease Control and Prevention; COPD: chronic obstructive pulmonary disease; CAD: coronary heart disease; HIV: human immunodeficiency virus; CKD: chronic kidney disease; CHF: congestive heart failure; SUD: substance use disorder; SMI: serious mental illness; PTSD: post-traumatic stress disorder; VA: Veterans Health Administration; CBOC: VA community-based outpatient clinic

© 2024 Hebert PL et al. *JAMA Network Open.*
