# Supplementary material for: Changes in Outpatient Health Care Use After COVID-19 Infection Among Veterans
Source: JAMA Netw Open. 2024 Feb 9;7(2):e2355387. doi: 10.1001/jamanetworkopen.2023.55387 (PMC10858406; doi:10.1001/jamanetworkopen.2023.55387)
Supplement: Supplement 3. — Data Sharing Statement [file jamanetwopen-e2355387-s003.pdf]

Data Sharing Statement

Hebert. Changes in Outpatient Health Care Use After COVID-19 Infection Among Veterans. *JAMA Netw Open*. Published February 09, 2024. doi:10.1001/jamanetworkopen.2023.55387

Data
Data available: No

Additional Information
Explanation for why data not available: VA HSR&D does not allow data sharing outside of the VA firewall and without appropriate approvals
